# Hæmorrhagia, or Hæmorrhage

**Published:** 1839-01-12

**Authors:** N. Chapman

**Affiliations:** Professor of the Theory and Practice of Physic, in the University of Pennsylvania


### MEDICAL EXAMINER.

DEVOTED TO MEDICINE, SURGERY, AND THE COLLATERAL SCIENCES.

No. 2.] PHILADELPHIA, SATURDAY, JANUARY 12, 1839. [Vol. II.

### HÆMORRHAGIA, OR HÆMORRHAGE.

*By* N. Chapman, M. D., *Professor of the Theory and Practice of Physic, in the University of Pennsylvania.*

(Continued from page 9.)

The usual objection raised against this theory of its being scarcely conceivable that so copious a discharge as is sometimes witnessed, should proceed merely from exhalation, I do not think has much force. Even from a very limited point the effusion may be very profuse. As one, among many other examples of it, epistaxis suggests itself, where enormous quantities of blood, on some occasions, proceed from a very small portion of the lining of a nostril. Further,—and with this additional illustration I must be content,—not many years ago, I attended, with Dr. Dewees, a young man, otherwise in good health, who, for three successive days, lost about three pints of blood daily from his gums. Both these and his teeth were remarkably sound, to all appearance. By wiping the gums clean, the blood was seen, in a moment, oozing out from numerous pores.

What takes place in the exhalents, to which this effect is to be referred, we do not know. To say, in the language of Bichat, that it is owing "to a change in the activity of the capillaries, as well as in the specific sensibility of the exhalents," were to repeat a phrase, meaning no more, than that they are in a morbid condition, preventive of the performance of their natural functions, which is a mere truism. It is probable, the phenomenon is referrible to some irregular operation of the nervous system, by which there is a deficiency of innervation. Many of the circumstances of hæmorrhage sustain this view, which might, indeed, be very plausibly vindicated, had I time to dwell on such speculations. Destitute, however, as we may be, of any precise information on this point, it is still, perhaps, not more obscure than many other parts of pathology. The extreme vessels in a healthy state, exercise the function of secreting and throwing out a mucous, serous, or some more attenuated fluid, according to their respective offices. Becoming diseased, this capacity is sometimes utterly lost, and blood which enters them for the elaboration of these fluids, passes through unaltered. As an illustration of this position, the case of menorrhagia is exceedingly striking. The uterine capillaries, in health, by a secretory action, convert the blood into a peculiar fluid, denominated menses. In certain conditions, however, they are deprived of this faculty, and pure blood escapes, constituting a real hæmorrhage. Lientery, also, affords an analogy, which, though remote, is still pertinent. Food received into the stomach, in this affection, owing to a singularly irritable condition of the alimentary canal, is hurried down, and evacuated without any alteration from the digestive operations.

The hypothesis, under view, is further rendered probable by the circumstance of the blood, in some cases, being partially changed, a sufficiency of it only remaining to colour the mucus, or other secreted fluid. We meet with such appearances in hæmoptysis, and still more frequently in dysentery. The evacuations in this disease, which exhibit every variation from pure mucus to nearly pure blood, surely can only be accounted for on such a supposition. More explicitly stated, these various aspects of the stools, are owing either to gradations of diseased action in the vessels of the same portion of the intestine, or while those of one part may be secreting mucus, another is extravasating blood, which, mixed together, present the complicated character described.

In a word, the doctrine I have endeavoured to expound supposes, that vital hæmorrhage is an effusion from the exhalents of some of the elementary tissues, and not at all occasioned by rupture of the large vessels entering into the composition of these, or the substance of the organs. Even in cases where blood is met with in the latter situations, as in cerebral and pulmonary apoplexy, it is referrible mostly to the same sort of exhalation.

Of all the hæmorrhages, those of the brain are most apt, it is said, to be induced by rupture, which is attempted to be explained, as well by the peculiarity in the conformation and arrangement of its vessels, as their greater liability to disease. Granting this, still such events are comparatively rare.

To insist on the doctrine of exhalation, I have been led the more strenuously,—for though now demonstrated to be true, from long and general usage, a phraseology continues to be employed by the profession, and others, that warrants a different conclusion. As much almost as formerly, do we hear of the rupture, bursting, or breaking of blood-vessels, in connexion with the occurrence of hæmorrhage, which is inaccurate in itself, conveying a mistaken pathological notion, and, among other evil consequences, is well calculated to create unnecessary alarm.

Except, perhaps, the purely fibrous, every tissue is subject to sanguineous effusion.

But in the mucous, in all its distributions, though especially in the lining of the alimentary and pulmonary passages, it chiefly takes place, and which may be accounted for, as well from the greater vascularity of this texture, as by its freer exposure to morbid agencies. Each one of the more common hæmorrhages, epistaxis, hæmoptysis, hæmatemesis, hæmorrhois, the vesical and the uterine, in the unimpregnated state of the organ, belong to this tissue, and are thus induced. Those of the cellular membrane are usually exhibited in the substance of organs into which it enters as an interstitial tissue, though sometimes, in the sub-cutaneous, and other diffused portions of it, while the dermoid usually effuses in the shape of petechiæ or vibices, or what is called hæmorrhea purpurea, from its greater extent, amounting sometimes to real hæmorrhage. Blood sometimes, however, oozes through the pores of the skin in place of the serous fluid in the perspiratory process. Both Aristotle and Theophrastus notice the occurrence, and I formerly cited a passage from Lucan in which it is described. It has been met with in low fevers, as stated by Huxham and other writers, though more generally seems to have been produced by extreme mental or bodily anguish, or the two united, or whatever, indeed, throws the nervous system into vehement commotions. Examples without number, might be collected to this purpose, some of the most striking of which I shall give. Charles IX. of France, a cruel and infamous sovereign, tortured by remorse on the approach of death, thus perished. Mezeray, the historian, who furnished this fact, also mentions that of a commander of a garrison, who, condemned to die for having cowardly surrendered it, broke out with such a sweat, as soon as he saw the gallows on which he was to be executed. Lombard tells us of a general similarly affected by the mortification or dread of the consequences of losing a battle,—and of a woman, at the terror of robbers, into whose hands she had fallen. We have from Fabricius, the instance of a mother, who, in the depth of grief for the loss of an only son, thought that she beheld his apparition, imploring her prayers for his release from purgatory, became violently agitated, and was seized with this cutaneous exudation. The Saviour of man himself, in the moment of his most excruciating sufferings, presented this heart-rending spectacle. "By thy agony and bloody sweat," is, indeed, one of the touching invocations addressed to his mercy, in the litany of the Church.

That hæmorrhage is also incident to the serous membranes, the arrachoid, the pleura, the pericardium, and peritoneum, we are assured by extravasated blood having been found in their respective cavities, or intervening spaces.

Formerly, hæmorrhage was divided into active and passive; and on this pathological distinction, a controversy is now maintained, in which the latter state is utterly denied by one party. This is really a dispute of definition, different meanings being attached to the terms employed. The term passive, I am inclined to believe, was adopted, conventionally, to signify not an absolute want of action, as it strictly imports, but a weaker state, in contradistinction to that activity which belongs to febrile hæmorrhage. Conceding, that topical irritation and congestion may exist with general weakness, and this is all, which, perhaps, will be demanded, I must still insist, that extravasations of blood do take place, though scarcely to be considered as genuine hæmorrhage, in a state in which there is neither congestion nor action in the vessels concerned. It is usually the result, as formerly intimated, of the feebleness of vital power, affecting both the vessels and the blood, the one being very relaxed, and the other thin, or wanting in consistency,—proofs of which are exhibited in various diseases of exhaustion. As, after death, where we often meet with large livid patches on the surface, from sanguineous exudations, as well as collections of blood within the cavities, which, as regards the former, we have occular demonstration that they did not pre-exist, so does it happen, in the last expiring efforts of life, under the circumstances stated.

The language of Andral, who is among the very highest authorities, is very conclusive on this point. "The existence," he says, "of vascular congestion is not essential to the production of every species of hæmorrhage. It is sufficient that the qualities of the blood should be so modified, that its molcules lose their natural form of cohesion, in which case the blood escapes from its vessels with the greatest facility, and hæmorrhages occur at the same moment in different parts of the body, totally unconnected with the presence of any irritative or inflammatory action. Examples of such hæmorrhages are supplied in scurvy, in typhus, and other diseases, in which there is a certainty that the blood has undergone such changes. How the vessels are modified, so as to permit their contents to escape, is a mystery which we cannot divine. But so much is ascertained, that the blood, so far from accumulating in them, constituting congestion, is permitted to flow out as fast as it arrives."

As illustrating this principle, he adduces the analogies of the profuse colliquative perspirations, in states of extreme weakness, and also the cold sweats of death, to which he might have added some instances of hydropic effusions.

To close, however this controversy, let these different theoretic views be subjected to practice, the fairest, and indeed the only test of speculative truth. Do we in any such case of feeble or passive hæmorrhage, resort to evacuant or depletory means? But, on the contrary, are not all our remedies of a tonic or even stimulating character to restore tone and impart vigour to the relaxed vessels and general system—and subsequently to tonics, to change and improve the character of the blood itself? To such an ordeal do I always bring a theory. Before adopting it, I must see how it works in a sick room.

An opinion at one time was very generally entertained, that in early life, hæmorrhage proceeds from the arteries, and afterwards from the veins, plethora then being transferred to the latter portion of the circulatory system. But some dissented from it, and maintained, that at every stage of existence, it is mostly venous, when coming from the hepatic, splenetic, gastro-enteric or hæmorrhoidal vessels—while that from the nasal, pulmonary and uterine, as uniformly to issue from the arteries. Excepting malœna, and even this can scarcely be considered as an exception, since the portal circulation is not strictly venous, it seems to me quite certain that all genuine hæmorrhages, are of an arterial nature. Distinct from other evidence to be hereafter cited when treating of the separate hæmorrhages, such a conclusion seems to me irresistible, from the contemplation only of the mode in which the effusion takes place, it being through the capillaries performing the secretory and nutritive offices, which are a part of the arterial and not of the venous system.

Coming to the practical portion of my subject, I am met, at the very threshold, by the question, whether it is expedient in any case of hæmorrhage, to interfere, or whether at all times it should not be left to the uncontrolled efforts of nature?

It was a doctrine originally advanced by Stahl, in which he was followed by his disciples, and some later authorities, that these discharges are designed to remove a dangerous repletion of system, which being sufficiently effected, they spontaneously cease. That such a view, with certain limitations, is correct, cannot be denied. Most hæmorrhages of an active character are undoubtedly salutary. It is also true, that the sudden checking of the nasal and hæmorrhoidal discharges is dangerous in a disposition to cerebral affection, as well as in fever, and many other acute and chronic diseases. Nor can it be disputed, that the flow of blood is often duly suppressed by the natural resources. But admitting these postulates, it will still appear that we cannot uniformly confide to nature the charge of such cases. Efficient and wise, sometimes, in her endeavours, she is oftener the reverse, and we are constrained, to prevent evil, to take the management out of her hands. As an example, she frequently neglects, or is not able, to give to these discharges a proper direction, and instead of blood issuing from the nasal or rectal vessels, it is poured into the cranial or some other occluded cavity, from which it cannot escape or be removed. Not less is her blindness or incompetency evinced, in resorting, at all, to the expedient, in enfeebled, or in permitting an excessive expenditure of blood, in other states of the system. The hæmorrhage, too, being copious, she cannot always by syncope, or by any other means, afford relief. These, then, are the circumstances demanding the interposition of art, and without which, indeed, in numerous instances, the event must be inevitably fatal.

The leading indication in all inordinately profuse hæmorrhages, is to suppress the flow of blood, and when they are active and febrile it is done.

1st. By reducing the force of vascular action by evacuations, and especially by bleeding, general and topical.

2d. By what are termed refrigerants, which may be external or internal, the one consisting of cold applications, and the other of a set of medicines, so called, as nitrate of potash, &c.

3d. By the sedative articles, or such as are presumed to abate the energies of the moving powers of the circulation, without any evacuation, as digitalis, &c.

4th. By constringing the mouths of the vessels. Whether there be a medicine with such a property, is to me exceedingly problematical, and perhaps does not exist. Yet it is supposed that we are in possession of many, as certain preparations of lead, of copper, of zinc, of alum, the mineral acids, and creosote, besides several from the vegetable kingdom, as tannin, or those substances containing this principle. Directly applied to the vessels, some of these are styptics—though acting through the medium of the system, they probably have no such effect.

5th. Causing a revulsion in the circulating fluids, from the affected part to one less interesting to the animal economy, is another principle in the cure of hæmorrhages, which occasionally succeeds, where the means are judiciously selected and well timed. It is customary to recur to stimulating pediluvia, or sinapisms, or blisters to the extremities, with this intention.

Excepting that evacuations, particularly by venesection, are to be more limited, or sometimes entirely excluded, I am not aware that there is any material difference in the means of meeting the same indication in the less active hæmorrhages. Emetics, however, are here undoubtedly useful.

To prevent the recurrence of the affection, by guarding against the exciting causes, and removing the pathological condition, which disposes to its production, is the second indication. The latter may be found in some positive lesion of organs, requiring a distinct mode of treatment, accommodated to the nature of the case. But owing merely to the hæmorrhagic diathesis or tendency, the most effectual measure, perhaps, consists in such diet as is the least calculated to fill the vessels with blood or excite their movements. Care, at the same time, should be observed, to anticipate the recurrence of the hæmorrhage where it is menaced, by a renewal of the means of direct reduction.

In the weaker state of the affection, we must endeavour to invigorate the system and equalise the circulation, by the well regulated use of tonics, particularly the martial preparations, and by a course of living co-operating to the same end, without, however, its having any heating or stimulating effect. Even in these cases, local irritations or congestions must be watched, and timely removed, by topical bleeding or vesication.

Much is to be expected from exercise, as an auxiliary to diet, in the prevention of each state of hæmorrhage. Eminently has it the power of promoting the secretions and excretions, of renovating healthy action, and especially of re-establishing a just equilibrium in the circulation, thereby obviating those local accumulations which prove the proximate cause of the effusion. Every thing else failing, an alterative course of mercury, very cautiously conducted, has often succeeded in both forms of the disease, acting as well by the restoration of healthy secretory power, as removing visceral obstructions, constituting the remote sources of the affection.

In regard to the purely passive hæmorrhages, I have only to say, that as incidents of a very low and depraved condition of system, the treatment of them essentially consists in the correction of the general vitiation from which they proceed. It may, however, be added, that merely to check a flow of blood, the phosphorus, the spirits of turpentine, and creosote, are thought to be singularly well adapted, though it is with the turpentine only that I have any experience.

As it usually appears, I think that too much importance is by many attached, in the management of vital hæmorrhage, to its suppression. Great alarm is created by it in the individual himself, as well as in his friends, and from which the medical attendant is not always entirely exempt. Every exertion is therefore made to check it, and this being accomplished, the anxiety which previously existed, heedlessly subsides. Lulled into false security, the patient is too often permitted to revert to his former habits, without any permanent plan of treatment, till again awakened to a sense of danger by a repetition of an attack, and in this way, he proceeds till the complaint is often irremediably fixed. Now, the hæmorrhage in itself is comparatively of little moment, for the most part, indeed, beneficial, and the real object of attention should be the correction of the condition giving rise to it, and which, by neglect, in numerous instances, leads to the most disastrous consequences.

